# Fracture resistance and mode of failure of CAD/CAM milled versus 3D-printed resin composite inlays: an in vitro study

**DOI:** 10.1186/s12903-026-07975-7

**Published:** 2026-03-21

**Authors:** Mostafa Nasser Abdel-moniem, Ola Mohammed Ibrahim Fahmy, Rehab Khalil Safy

**Affiliations:** 1 Department of Conservative Dentistry, Faculty of Dentistry, Suez Canal University, Ismailia, Egypt; 2 Department of Conservative Dentistry, Faculty of Oral and Dental Medicine Dentistry, Misr International University, Cairo, Egypt

**Keywords:** 3D printing, CAD/CAM milling, MOD inlay, Fracture resistance, Failure mode

## Abstract

**Objective:**

This study investigated the fracture resistance and mode of failure of CAD/CAM milled versus 3D printed resin composite inlays under the influence of thermocycling.

**Materials and methods:**

Brilliant Crios CAD/CAM milled and Crowntec 3D printed resin composite materials were used in this study. Twenty sound freshly extracted human mandibular third molars were selected for this study and randomly divided based on manufacturing technique into two groups: CAD/CAM milled, and 3D printed resin composite inlays. A universal testing machine was used to estimate the fracture resistance, and the mode of failure was investigated for fractured specimens. Inferential statistics for evaluating and comparing milling and 3D-printed manufacturing techniques were carried out via independent samples t tests at the 0.05 significance level.

**Results:**

While the 3D printing group recorded numerically higher fracture resistance, no statistically significant difference was found (*p* > 0.05). Evaluating the mode of failure of the CAD/CAM milling group specimens revealed that there was no significant difference between all the recorded failure modes (*P* > 0.05). On the other hand, a significant difference was revealed between the different recorded modes of failure in the 3D printing group (*P* < 0.05).

**Conclusions:**

Both 3D-printed, and CAD/CAM milled resin inlays had comparable fracture resistance that exceeded the physiological masticatory force in molar region; however, most of the failure modes recorded for both materials were irreparable failures involving more than half of the tooth and extending below the CEJ.

**Clinical significance:**

3D printed resin composite material is a promising option in fabrication of indirect restorations with lower cost and more time saving. Long term clinical studies are recommended to evaluate clinical performance of 3D printed resin composite materials.

## Introduction

Dental practitioners face great challenges in restoring vital posterior teeth that are badly broken down. Restorative procedures often compromise tooth continuity, increasing susceptibility to fractures particularly when using direct techniques [1]. Recent literature suggested a strong relationship between the remaining amount of tooth strictures and the restoration survival [2]. It was reported that the risk of annual failure rate (AFR) increased by 30% to 40% for each additional missing wall [3]. Also, in posterior teeth with fewer than two remaining walls, resin composite restorations failed 3.3 times more often compared to those with four walls [4]. Therefore, indirect restorations have been introduced to rehabilitate badly decayed or fractured posterior teeth [5]. Among these indirect restorations, the inlay cavity is a minimally invasive cavity that has the advantages of preserving tooth structure and improving stress distribution. However, unrepairable fractures were noticeably greater with MOD inlays with mixed types of failure [6].

The introduction of computer aided design/computer aided manufacturing (CAD/CAM) in the field of restorative dentistry has revolutionized the techniques of fabricating indirect restorations and becoming popular alternatives to conventional techniques [7]. CAD/CAM milling technology has enabled the use of resin composite blocks that provide superior properties, such as improved composition and a better degree of conversion, under ultrahigh temperature and high pressure [8]. Additionally, compared with ceramics, resin composite blocks have a lower hardness, which results in a reduced wear of opposing natural teeth and better bonding to resin cement. As all resin composite blocks exhibit lower brittleness, they are also less prone to chipping during manufacturing or catastrophic failure during service [9]. 

Although the CAD/CAM milling technique is still the gold standard for the digital CAM process [10], several drawbacks are associated with this technique such as wear of the milling bur, time and material wastage, and greater possibility of defects due to the poor machinability of high-strength blocks [11]. More recently, 3D printing fabrication techniques have gained popularity. The printing process takes place layer by layer allowing an almost unlimited variety of shapes and levels of complexity, in addition to material and time saving that can’t be achieved with the milling technique [12].

Notably, fracture is regarded as the most common cause for replacing indirect restorations. Fracture resistance of restorations and their ability to withstand masticatory forces ranged from 500 to 900 N in the molar region [13] are crucial factors in the long-term viability of restorations [14, 15]. Several studies were carried out recently concerning the different comparisons in mechanical properties between CAD/CAM and 3D printed resin composite materials [16–19]. Although these studies suggested high durability of 3D printed resin composite materials and comparable results between CAD/CAM milled and 3D printed resin composite crowns, a limited number of studies were found discussing fracture load related to inlay cavity designs [20]. Therefore, the aim of this study was to evaluate fracture resistance and mode of failure of CAD/CAM milled versus 3D printed resin composite inlays after thermocycling simulation of six months clinical situation. The null hypothesis of the current study is that there is no significant difference in fracture load and mode of failure between the CAD/CAM and 3D printed resin composite inlays after thermocycling.

## Materials and methods

The current study was performed after being waived by the Research Ethical Committee of the Faculty of Dentistry, Suez Canal University, Egypt, (No. 583/2022).

### Sample size calculation

A minimum total sample size of 16 samples (*n* = 8) was sufficient to detect the effect size of 0.386, at a power (1-β = 0.90) of 90%, at a significant probability level of *p*< 0.05 and a partial eta squared of 0.13 [21, 22]. G*Power software version 3.1.9.6 was utilized to determine the sample size. A total sample size of 20 samples (*n* = 10) was used in this study to overcome the risk of pretest failure and statistical outliers; this sample size agreed with previous study [16].

### Teeth selection

Twenty sound human mandibular freshly extracted impacted third molars were selected for the current study. The inclusion criteria of selected teeth were sound mandibular third molars with approximately similar buccolingual and mesiodistal dimensions. Teeth with cracks, fracture lines or any developmental anomalies were excluded [23, 24]. As soon as they were extracted, selected teeth were thoroughly cleaned under running water, and tissue remnants on the teeth’ surface were removed with an ultrasonic scaler [25]. The buccolingual and mesiodistal dimensions of selected teeth were measured via a digital caliper (Mitutoyo Corp., Tokyo, Japan) [26]. The teeth with approximately buccolingual and mesiodistal dimensions of 9 ± 1 and 10 ± 1 mm respectively were included in the study, whereas teeth with any other dimensions were replaced. Afterward, the selected teeth were preserved in a 0.1% thymol-containing isotonic saline solution at room temperature for no more than three months [27].

### Teeth mounting and periodontal ligament simulation

**Fig. 1:**
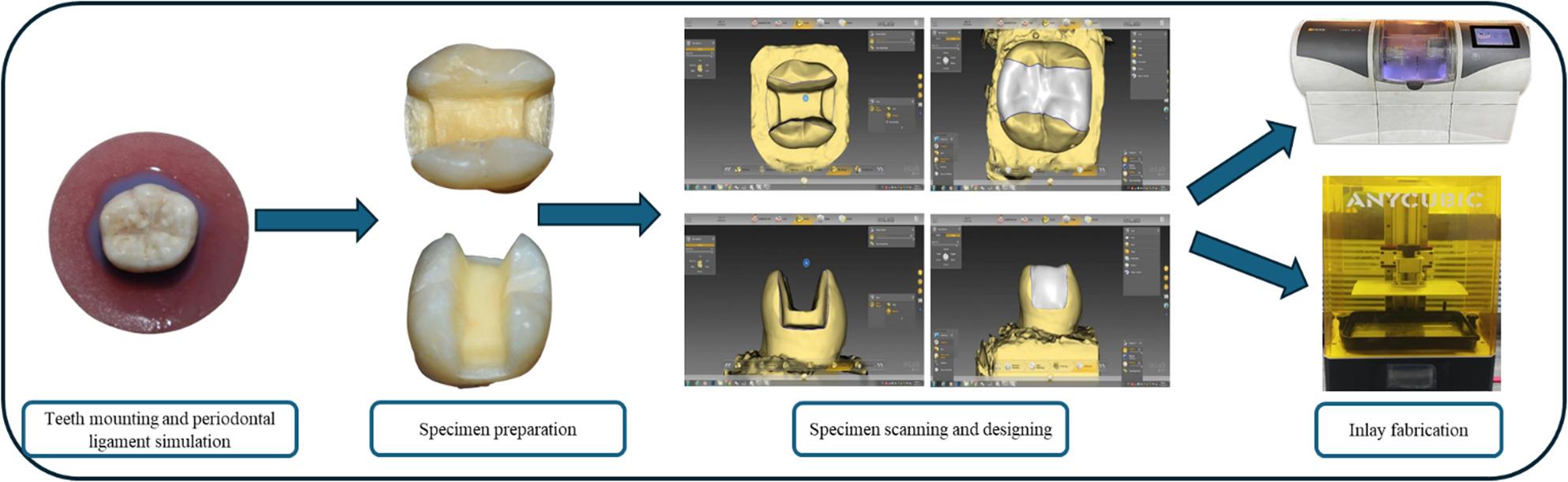
Schematic figure (tooth mounting, specimen cavity preparation, scanning and designing, and inlay fabrication)

Before the experiment, the teeth were embedded in molten wax 1–2 mm below the cementoenamel junction (CEJ), which acted as a spacer. A rectangular silicone mold of 2.5 × 2.5 cm was used for the fabrication of acrylic bases of self-curing polymethyl methacrylate resin. Afterwards, waxed teeth were embedded 1–2 mm below the CEJ in the acrylic bases during the dough stage. After acrylic bases were completely cured, they were merged in boiled water for wax melting. Then, a 1 mm thick layer of light polyvinyl silicone impression material (Ghenesyl, Lascod, Italy) was applied in the space created by molten wax for periodontal ligament simulation to simulate the stress distribution generated over teeth to be as close as possible to the clinical situation [20]. Each acrylic block was labelled with a non-repeated number from one to twenty via a permanent marker (Fig. 1).

### Inlay cavity preparation

All mounted molars were subjected to standardized mesio-occluso-distal (MOD) cavity design following the general guidelines for aesthetic inlay restorations [28]. A high-speed hand piece was used to prepare each cavity under copious water-cooling via a cylinder flat-ended diamond bur (SF-41, ISO 109/010, Mani, Japan) that was replaced every five preparations. The depth of the pulpal floor was 3 mm from the occlusal cavo-surface margin, and the remaining buccal and lingual wall thickness was adjusted to 2 mm via a digital caliper. Considering the proximal box, the occluso-gingival depth was approximately 4.5 mm from the occlusal cavo-surface margin, the gingival margin was placed 1 mm just above the CEJ, and the axial wall width was 1.5 mm mesiodistally. All the prepared cavity walls were nearly parallel to the long axis of the tooth with butt joints cavo-surface margins and rounded internal lines and point angles [24]. Each prepared cavity dimensions was checked via a periodontal probe and a digital caliper (Fig. 1).

### Specimens’ randomization and grouping

All the prepared specimens were equally and randomly divided into two groups based on inlay manufacturing technique (*n* = 10). In group (M1): the cavities were restored by milled CAD/CAM resin composite blocks (Brilliant Crios^®^, Clotene, Switzerland) whereas in group (M2): the cavities were restored by 3D printed resin composite (Crowntec^®^, Saremco, Switzerland) as shown in Table 1. The randomization was performed via the website http:/www.randomizer.org, after which the specimen blocks were labelled.

**Table 1:** Materials’ brand names, descriptions, compositions, manufacturers, and batch numbers

Group	Materials’ brand name	Description	Composition	Manufacturer	Batch number
M_1_	Brilliant Crios^®^	CAD/CAM resin composite block	Crosslinked methacrylates (Bis-GMA, Bis-EMA, TEGDMA). Barium glass (*<* 1 μm) and amorphous silica (SiO2;*<*20 nm) 70.7 wt%	Coltène, Whaledent AG, Altstätten, Switzerland	L29361
M_2_	Crowntec^®^	3D printed resin composite	4,4-isopropylidiphenol, ethoxylated and 2 methylprop-2enoic acid; (Bis-EMA), TPO UV photo initiators. Silanized dental glass and pyrogenic silica (0.7 μm) 30–50 wt%.	SAREMCO Dental AG, Rebstein, Switzerland	E522

### Scanning and designing

Each prepared cavity was scanned by an intra-oral scanner (Primescan, Dentsply, Sirona, Germany), and the scanned files corresponding to labels on each acrylic block were labelled. Scanned specimens were used for designing MOD inlay restorations using CEREC software version 4.5.1 with specified parameters and cement space of 50 μm starting at 1 mm away from the margins. For the CAD/CAM milling group (*n* = 10), the design files were transmitted through a wireless network (closed system) to the milling machine (CEREC MC XL, Dentsply, Sirona, Germany). The 3D printed group (*n* = 10) design files were exported to a Standard Tessellation Language (STL) file, stored on a flash memory and sent to a 3D printing machine (Anycubic photon mono X 6 K, Hong Kong Anycubic Technology Co., Ltd, China) (Fig. 1).

### CAD/CAM milled inlays fabrication

Ten milled inlays were fabricated from Brilliant Crios CAD/CAM resin composite blocks. A new set of step burs and extra fine cylinder burs (12 S and 12 EF, Dentsply, Sirona, Germany) were used for the milling of the ten blocks according to the manufacturer’s instructions. At the end of the milling process, the remaining part of the block was removed, and a cut-off wheel (Keystone Industries, USA) was used for gently smoothing the external surface of all milled inlays with optical magnification followed by 10 min. of ultrasonic cleaning in distilled water [29]. Each milled inlay was subsequently seated on its corresponding MOD cavity, secured via elastics and stored in distilled water (Fig. 1).

### 3d printed inlays fabrication

The remaining ten inlays were fabricated from Crowntec permanent 3D printed resin composite. All 3D printed inlays were fabricated through standardized printing parameters, where 50 μm layer thickness and horizontal 0º building angle were used according to the manufacturer’s instructions. At the end of the printing process, a 96% ethanol-soaked cloth and micro-brushes were used for 5 min. to get rid of residual resin monomers at the external and internal surfaces of all the 3D printed inlays, which were then dried thoroughly with an air syringe. For complete polymerization, the 3D printed inlays were subsequently placed in post-curing unit (Anycubic wash and cure plus, Hong Kong Anycubic Technology Co., Ltd, China) which rotated while they were exposed to 405 nm. UV lights for 6 min. Finally, 50 μm glass beads were used for air abrasion of the external surfaces at 1.5 bar, then supports that held the inlays to the 3D printer platform during printing were removed with cut-off wheel [29]. Afterwards, each 3D printed inlay was seated on its corresponding MOD cavity, secured and stored as mentioned for CAD/CAM milled cavities (Fig. 1).

## Cementation of inlays and thermocycling procedure

For the cementation procedure, the fitting surfaces of all the inlays were sandblasted with 50 μm aluminum oxide (Al_2_O_3_) powder (Jeep Dental, Germany), from a distance of 10 mm for 10 s with 2 bar pressure via air abrasion sandblaster gun (AZDENT, China) according to the manufacturer’s instructions to improve the bonding of the adhesive resin cement. All the restorations and prepared cavities were then thoroughly rinsed and gently air dried for 2 s with oil-free air. Immediately after all specimens were cleaned, self-adhesive resin cement (Panavia SA Universal, Kuraray, Japan) was applied directly to the restoration’s fitting surface and the prepared cavity. Afterwards, each specimen was fixed in its exact position during cementation via a customized locking device that provides a standard load. Initial photopolymerization was performed for three seconds from each surface (occlusal, mesial and distal) using a LED light unit (I-LED, Woodpecker, China) at 1,200 mW/cm2. Any excess cement was carefully removed with a micro-brush and further cured for 40 s from each surface. Each specimen was left under the clamp of the customized locking device after photopolymerizing the resin cement for 10 min [30].

Afterwards, all specimens were subjected to thermocycling protocol with a thermocycling machine (Thermocycler 1100, SD-Mechatronik, Westerham, Germany) for 5000 cycles in water baths between 5 °C and 55 °C with a dwell time of 30 s at each temperature, with transfer time of 5 s from one bath to another to simulate six months of clinical service in the oral environment [31, 32]. All the previously mentioned procedures were carried out by the same operator to ensure consistency and reduce variability.

### Evaluation of fracture resistance and mode of failure

Fracture resistance was the primary endpoint in most studies because it directly measures the force a restoration can withstand before fracture. A universal testing machine (Instron model 3345, England) was used to assess the fracture resistance of each specimen. Each mounted specimen was attached to the lower fixed head of the machine. The load was applied by the upper movable head with a 5 mm round stainless-steel head at a crosshead speed of 1 mm/min. up to specimen failure. The load was applied vertically to the long axis and centered on the occlusal plane at the central fossa [17]. A tiny aluminum foil was inserted between the metal sphere and the occlusal surface of the inlay to ensure an even load distribution. The load required for failure was verified by software (BlueHill Universal Instron, England), and the maximum load to fracture was recorded in Newtons (N) [19]. Immediately after the fracture resistance test, the failure mode of each specimen via direct examination with magnifying loupes and classified according to the Burke index [33] into three repairable and one non-repairable failure. The type I repairable failure was distinguished by isolated fracture of the restoration, while in type (II) restoration fracture involved a small tooth portion. If the fracture involved more than half of the tooth above the CEJ, it was classified as type (III). Non-repairable failure (type IV) was denoted for fracture involving more than half of the tooth and below the CEJ [34, 35].

### Statistical analysis

Following data collection, the data were examined, revised, and organized in tables and figures in terms of the means and standard deviation via Microsoft Excel 2016. Outlier detection and normality statistical tests were performed to determine whether the data were parametric or nonparametric. Both graphical and numerical descriptions were used in the descriptive statistical analysis of the data. Inferential statistics for evaluating and comparing milling and 3D-printed manufacturing techniques were carried out by independent samples t tests at the 0.05 significance level. Statistical Package for Social Science SPSS (IBM-SPSS ver. 28.0 for Mac OS) computer software was used for data analyses.

## Results

The data in Table (2) show that the highest load value was recorded in the 3D printing group (2059.9 ± 369.0 N), whereas the lowest value was recorded in the CAD/CAM milling group (1867.2 ± 363.6 N), with no significant difference between them (*p* = 0.255).

**Table 2:** Fracture resistance for the milling and 3D printing groups presented as minimum, maximum, mean and standard deviation (Mean ±SD)

Descriptive statistics	Fracture resistance values (N)		independent t-test p-value
	Milling	3D Printing	
Min.	1484.8	1641.0	0.255 ns
Max.	2391.8	2562.7	
Mean	1867.2	2059.9	
SD	363.6	369.0	
Mean ±SD	1867.2±363.6	2059.9±369.0	

**Fig. 2:**
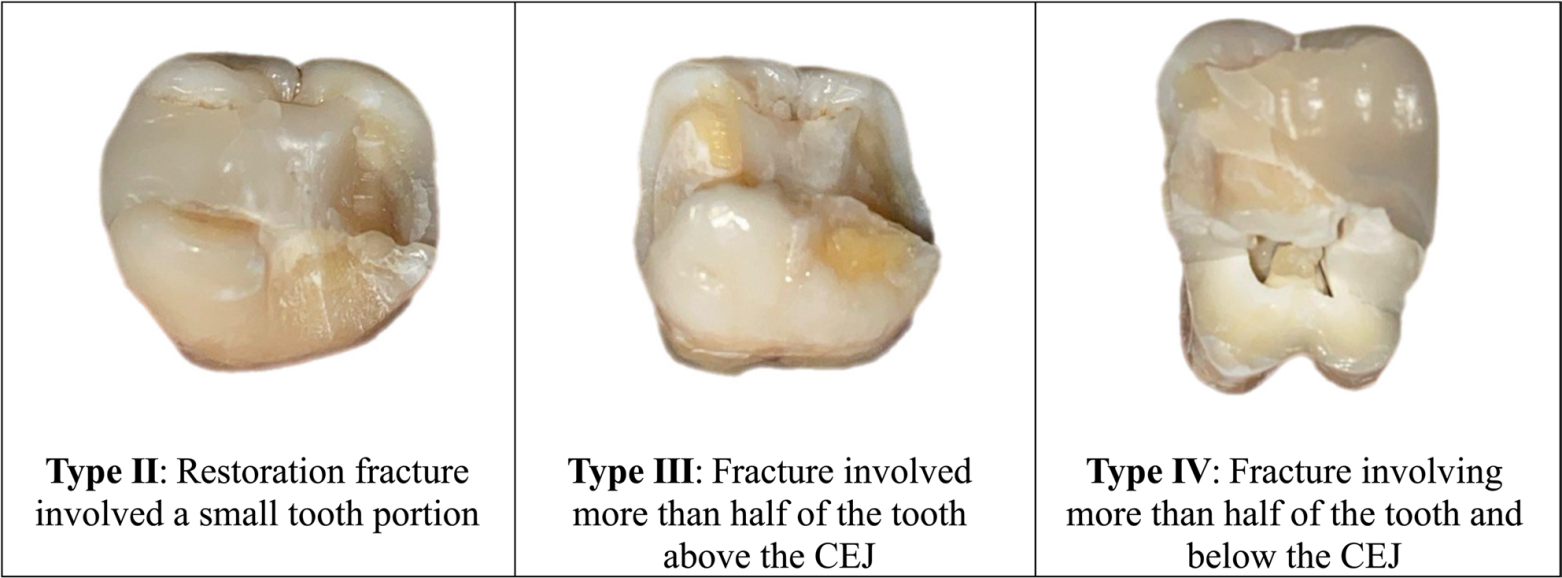
Failure modes

Evaluating the mode of failure of all the tested specimens in the CAD/CAM milling group revealed that 20% of the specimens exhibited type II repairable failure (*n* = 2), 30% of the specimens exhibited type III repairable failure (*n* = 3), and non-repairable failure type IV was observed in the remaining 50% of the specimens (*n* = 5). The difference between all recorded failure modes in the CAD/CAM milling group was not significant (*P* = 0.497). On the other hand, the mode of failure in the 3D printing group included type II repairable failure in 20% of the specimens (*n* = 2), 10% of the specimens were type III (*n* = 1) and the remaining 70% of the specimens were recorded nonrepairable failure type IV (*n* = 7). A significant difference was revealed between the different recorded modes of failure in the 3D printing group (*P* = 0.045) (Table 3) (Fig. 2).

**Table 3:** Mode of failure frequency (n. and %) in the CAD/CAM milling and 3D printing groups

Mode of Failure	Mode of failure				Mann-Whitney test sign.
	Milling		3D Printed		
	n.	%	n.	%	
Type I (repairable)	0	0	0	0	0.491 ns
Type II (repairable)	2	20	2	20	
Type III (repairable)	3	30	1	10	
Type IV (non-repairable)	5	50	7	70	
Total	10	100	10	100	
Chi-square	0.497 ns		0.045*		

## Discussion

It is worth noting that fracture is considered the most common reason for replacing indirect restorations. Therefore, when considering the long-term longevity of the current restorations and their ability to withstand masticatory forces, fracture resistance test was performed [15–17]. This study investigated the fracture resistance and mode of failure of CAD/CAM milled versus 3D printed resin composite inlays under the influence of thermocycling. Both tested groups in the present study recorded fracture resistance values that exceeded the maximum masticatory force at the molar region (900 N) therefore are considered clinically acceptable [13]. Additionally, the results revealed that the fracture resistance of the 3D printing group was slightly greater than that of the milling group, with no significant difference. The lower fracture resistance of the CAD/CAM group may be attributed to the generation of different kinds of flaws detected along the thickness of the manufactured restoration that resulted from the milling burs. Consequently, fracture of the restoration may occur due to crack propagation in the restoration, which is initiated by flaws [6]. Moreover, microscopic grooves equivalent to the milling bur tip are produced on the inner surface of the milled restoration, with a subsequent increase in the cement space, resulting in an inferior fit that, in turn increases the risk of fracture [36]. In addition, vibrations and mechanical loads are induced during the milling process [37]. All of these risk factors in the previously mentioned scenario are not present in the case of 3D printed additive technology, in addition to the utilization of a higher resolution printer and controlled curing procedure in the fabrication procedure of the 3D-printed restorations in the current study, which might have resulted in increased cohesive strength and overall durability [38]. Notably, although the Crowntec 3D printed resin composite has a lower elastic modulus than the Brilliant Crios CAD/CAM material (4.5, 10.3 Gpa) respectively, which could be considered preferable in terms of the fracture resistance of the CAD/CAM material, this effect was minimized in the current study through utilization of 3 mm thickness of the 3D printed material and the use of self-adhesive resin cement as recommended by the manufacturer [39–41].

Thermocycling may have an adverse effect on the fracture resistance mean values of both groups. Both thermocycling and wet environments may cause chemical degradation of polymer matrix and hydrolysis of filler-matrix interfaces depending on the hydrophilicity of resin monomers and filler load of resin matrix. For the milling group, the hydrophilic nature of Bis-GMA and TEGDMA resin monomers may cause more degradation of the polymer network resulting in the development of microcracks in the milled specimens. The hydrophobic nature of the Bis-EMA resin monomer present in 3D-printed resin has been reported to reduce water sorption and enhance the fracture resistance of the resin because of its high molecular weight [42]. However hydrolytic degradation of the filler-matrix interfaces may be more common in the 3D printed specimens because of the lower filler load [43–45]. Hygroscopic expansion may cause internal expansion stress, that could result in microcracks or even cusp fractures in the restored tooth. Excessive water sorption may cause residual monomers, and ions to leak out because of material solubility resulting in increased void concentration and increased fracture potential [46].

These results come in agreement with *Donmez and Okutan*,* 2022* and *Diken Türksayar et al.*,* 2023* who recorded no significant difference in fracture resistance in between both the 3D printed and milled crowns [16, 19]. Meanwhile, these results disagreed with *Suksuphan et al.*,* 2024* who found significantly higher fracture resistance of nanoceramic, and polymer-infiltrated ceramics milled crowns when compared to 3D printed ones [17]. This argument may be due to utilization of CAD/CAM ceramic material instead of resin composite one.

Examining the failure mode of all tested specimens of the current study showed non significantly higher records of non-repairable failure type IV in the 3D printed group in comparison to the CAD/CAM one (70%, 50%) respectively. This may be related to the low elastic modulus of the 3D printed and milled resin composite in comparison to enamel [47]. It was reported that these resin materials transmit more stresses that exceed the intrinsic resistance of the surrounding tooth structures [48, 49]. Also, the increase in material thickness utilized in the current study (3 mm) seems to be inversely proportional to the repairability of fractures [40]. In addition to that, the MOD inlay cavity design was reported to have the lowest fracture resistance and the highest level of complex unrepairable fractures in comparison to the other indirect cavity designs [6, 35].

These results come in agreement with *Donmez and Okutan*,* 2022* who reported non-repairable fractures in both Crowntec 3D printed resin composite and Brilliant Crios CAD/CAM crowns [16]. Similarly, *Corbani et al.*,* 2020* observed higher frequency of nonrepairable failures with increased occlusal thickness (≥ 1.5 mm) of both 3D printed and milled restorations [40].

Thus, the null hypothesis of this study was accepted, as there was no significant difference in fracture resistance and mode of failure between the 3D printed and CAD/CAM milled inlays.

Finally, in light of the current study’s findings, it should be noted that some limitations that were encountered related to fracture resistance test in our study are the use of universal testing machine with a static manner and the lack of cyclic loading of the specimens before testing, which might change the outcomes. Therefore, using a chewing simulator and cyclic lading is recommended to obtain reliable results simulating the clinical condition.

## Conclusions

On the basis of the limitations of the current study, the following conclusions can be drawn:

- 1- Both 3D-printed and CAD/CAM milled inlays showed comparable fracture resistance values exceeding physiological molar loads. However, most failures were classified as irreparable.
- 2- Further studies to evaluate mechanical performance of the 3D printed resin composite materials are recommended.

## Data Availability

The datasets used and/or analysed during the current study are available from the corresponding author on reasonable request.
